# The effect of the endosymbiont *Wolbachia* on the behavior of insect hosts

**DOI:** 10.1111/1744-7917.12731

**Published:** 2019-11-11

**Authors:** Jie Bi, Yu‐Feng Wang

**Affiliations:** ^1^ School of Life Sciences, Hubei Key Laboratory of Genetic Regulation and Integrative Biology Central China Normal University Wuhan China

**Keywords:** aggression, insect hosts, learning and memory capacity, mating, sleep, *Wolbachia*

## Abstract

As one of the most successful intracellular symbiotic bacteria, *Wolbachia* can infect many arthropods and nematodes. *Wolbachia* infection usually affects the reproduction of their hosts to promote their own proliferation and transmission. Currently, most of the studies focus on the mechanisms of *Wolbachia* interactions with host reproduction. However, in addition to distribution in the reproductive tissues, *Wolbachia* also infect various somatic tissues of their hosts, including the brain. This raises the potential that *Wolbachia* may influence some somatic processes, such as behaviors in their hosts. So far, information about the effects of *Wolbachia* infection on host behavior is still very limited. The present review presents the current literature on different aspects of the influence of *Wolbachia* on various behaviors, including sleep, learning and memory, mating, feeding and aggression in their insect hosts. We then highlight ongoing scientific efforts in the field that need addressing to advance this field, which can have significant implications for further developing *Wolbachia* as environmentally friendly biocontrol agents to control insect‐borne diseases and agricultural pests.

## Introduction

*Wolbachia* is the most widespread group of intracellular bacteria belonging to the alpha‐proteobacteria. They can infect numerous of insects, isopods, spiders, as well as filarial nematodes (Werren, 1997; Zug & Hammerstein, 2012; Zhang *et al*., 2013; Turelli *et al*., 2018). *Wolbachia* are maternally transmitted and have evolved several strategies to manipulate host reproduction including parthenogenesis, feminization, male killing and cytoplasmic incompatibility to facilitate their own proliferation and transmission (Weeks & Breeuwer, 2001; Zheng *et al*., 2011a; Beckmann *et al*., 2017; LePage *et al*., 2017; Miyata *et al*., 2017; Harumoto *et al*., 2018). In the last decade, several lines of evidence have suggested that *Wolbachia* can be used as environmentally friendly biocontrol agents to control insect pest populations and disease vectors (McMeniman *et al*., 2009; Hoffmann *et al*., 2011; Moreira *et al*., 2011; Nguyen *et al*., 2015; Zhou & Li, 2016; Aliota *et al*., 2016a, 2016b; Garcia *et al*., 2019).

*Wolbachia* have been estimated to be the most ubiquitous endosymbiont, and can be used as an ideal model for studying the interactions between microbials and their hosts (Werren, 1997; Wu *et al*., 2016; Bhattacharya *et al*., 2017; Pan *et al*., 2018). So far, most studies on *Wolbachia* focus on the regulatory mechanisms by which *Wolbachia* mediate host reproduction (Zheng *et al*., 2011a, 2011b; Liu *et al*., 2014; Beckmann *et al*., 2017; LePage *et al*., 2017; Bonneau *et al*., 2018; Shropshire *et al*., 2018). However, it has been reported that *Wolbachia* distribute not only in the reproductive system, but also in various somatic tissues of their hosts where they might play a role in regulating host physiology and behavior (Casper‐Lindley *et al*., 2011; Albertson *et al*., 2013; Rohrscheib *et al*., 2015; Pietri *et al*., 2016). Symbiotic bacteria often alter the behavior of their hosts in various ways. Studies on arthropods that have horizontally acquired parasites show that behavioral changes may be induced by altered levels of gene expression in the central nervous system of the hosts (Thomas *et al*., 2005; Perrot‐Minnot & Cézilly, 2010). Although the precise mechanisms are not well understood, the behavioral change is inferred to be the consequence of selection acting on the parasite to promote the host's transmission. Behavioral changes are also found in hosts due to infection with vertically acquired intracellular bacteria such as *Wolbachia*. These modifications in host behavior may be caused by altered gene expression levels not only in the central nervous system but also in other places in the hosts (Richard, 2017; Bi *et al*., 2019; Schneider *et al*., 2019), and be driven by selection acting to promote bacterial transmission (Goodacre & Martin, 2012). This review presents recent advances in studies on the influences of *Wolbachia* infection on the behavior of their insect hosts and the current molecular mechanisms by which the endosymbiont modifies hosts’ behaviors.

## The distribution of *Wolbachia* in host somatic cells

Numerous researchers have documented that *Wolbachia* present not only in host gonads, but also in a wide array of somatic tissues of hosts. For instance, studies have demonstrated the presence of *Wolbachia* in the brains of *Drosophila melanogaster* and *D. simulans* (Min & Benzer, 1997; Dobson *et al*., 1999; Albertson *et al*., 2009; Albertson *et al*., 2013; Strunov *et al*., 2013), *Collembola* (springtails) (Czarnetzki & Tebbe, 2004), and *Eurema hecabe* (butterfly) (Narita *et al*., 2007). This indicates that *Wolbachia* have the potential to influence host behaviors. In addition to the nervous tissues, *Wolbachia* also infect many other somatic tissues such as the gut, salivary glands, fat body, hemocytes, and Malpighian tubules of their hosts where they may affect host immunity and behavior (Dobson *et al*., 1999; Casper‐Lindley *et al*., 2011; Albertson *et al*., 2013; Rohrscheib *et al*., 2015; Pietri *et al*., 2016; Pan *et al*., 2018; Saijo *et al*., 2018; Da Silva Gonçalves *et al*., 2019). Furthermore, *Wolbachia* has been found in muscle and wing tissue of some host species (Andersen *et al*., 2012). Pietri *et al*. (2016) have summarized the distribution of *Wolbachia* in different tissues of various hosts. Moreover, *Wolbachia* localization is variable. In *Drosophila*, it was revealed that the distribution of the pathogenic *Wolbachia* strain, *w*MelPop, in the nervous system of adults was temperature‐dependent: *Wolbachia* would move from the central brain to peripheral areas when temperature is increased (Strunov *et al*., 2013).

## The effect of *Wolbachia* on the sleep behavior of hosts

Sleep behavior in *Drosophila* has similar features to mammalian sleep, such as prolonged reversible inactivity, increased arousal thresholds and homeostatic influence (Hendricks *et al*., 2000; Shaw *et al*., 2000). The timing of sleep is controlled by a circadian system, which recurs approximately every 24 h in flies. Locomotor activity in *Drosophila* is organized in a 12 : 12 h light : dark cycle. *Drosophila* exhibits peaks of activity during dawn and dusk. Sleep homeostasis is traditionally measured by actively disrupting sleep, typically using mechanical stimuli. Sleep latency is the length of time to the first sleep bout after the arousing stimulus. When flies recover sleep after sleep loss, they do so by increasing its duration and/or by enhancing its intensity (Allada *et al*., 2017).

Currently, sleep in flies is normally monitored using the Drosophila Activity Monitoring System. An infrared beam directed through the midpoint of each tube (with inner diameter 5 mm and length 60 mm) measures an “activity event” each time a fly crosses the beam. Locomotor activity counts were collected every minute, and sleep was defined by zero activity counts for at least five consecutive minutes (Huber *et al*., 2004). By quantifying the average level of locomotor activity during 5‐min periods, one may compare locomotor activities of flies in different statuses when they are awake. Several studies have demonstrated that *Wolbachia* influence the sleep behavior of their hosts (Table 1). Albertson *et al*. (2013) once analyzed whether *Wolbachia* affected behavior and physiology, and showed a significant correlation of *Wolbachia* infection status with differences in sleep time during the day in *Drosophila*. Consistently, by testing the effect of *Wolbachia* infection on activity of *D. melanogaster* researchers revealed that *Wolbachia* infection decreased the percentage of time spent being active in both male and female flies, which suggests an increase in sleep time in flies of both sexes (Vale & Jardine, 2015). We recently found that *Wolbachia‐*infected flies increased sleep time in both male and female *D. melanogaster* when compared to the uninfected flies, which is due to the increased number of sleep bouts during nighttime (Bi *et al*., 2018).

**Table 1:** Effect of *Wolbachia* on the sleep behaviors of insect hosts

Sleep behavior	Effects	Reference
Sleep time	Changed sleep time during the day in *Drosophila*	Albertson *et al*., 2013
	Decreased the proportion of time active in *Drosophila* (suggesting increased sleep)	Vale & Jardine, 2015
	Increased the sleep time in *Drosophila*	Bi *et al*., 2018
Arousal threshold	Decreased the arousal threshold in *Drosophila*	Bi *et al*., 2018
	Increased the nocturnal activities (reflecting a decrease in arousal threshold and sleep disturbance) in *Drosophila*	Morioka *et al*., 2018
Sleep latency	Increased the sleep latency in *Drosophila*	Bi *et al*., 2018
Circadian rhythm and sleep homeostasis	Had no effects on the circadian rhythm and sleep homeostasis in *Drosophila*	Bi *et al*., 2018

*Wolbachia* infection led to a significant decrease in the arousal threshold of flies (Bi *et al*., 2018), meaning that *Wolbachia*‐infected flies are easily disturbed by a minimal stimulus relative to uninfected flies. This is consistent with the observation that *Wolbachia* infection resulted in an increase of the number of nighttime sleep bouts (Bi *et al*., 2018), indicating that *Wolbachia* infection could decrease the sleep quality in *D. melanogaster*. Morioka *et al*. (2018) also demonstrated that *D. melanogaster* carrying *Wolbachia* exhibited an increase in nocturnal activities relative to uninfected flies, which may reflect a decrease in arousal threshold during the nighttime. Moreover, we also found that *Wolbachia* infection caused an increase in sleep latency as the night progresses (Bi *et al*., 2018). This seems to be opposed to our common sense that arousal thresholds increase, and sleep latency decrease as the night progresses, which is how humans and other diurnally active animals behave. However, the overall pattern of the arousal threshold results is in accordance with previous observation in flies where arousal responses were lowest when flies are sleeping at maximal consolidated levels (Faville *et al*., 2015). In our study, flies showed peak sleep times between ZT 14–16 (ZT = zeitgeber time, where ZT 0 is lights‐on, and ZT 12 is lights off), which corresponded exactly to the times when flies showed the lowest responsiveness to an arousing stimulus and shortest sleep latency. Together, these results suggest that *Wolbachia*‐infected flies are incapable of experiencing deep and high‐quality sleep, or alternatively are more sensitive to the environment.

Complex interactions between circadian and homeostatic processes ensure that sleep occurs at right times in a day (Dijk & Czeisler, 1995). Plants and animals have these built‐in cycles which allow them to flower at the right time, sleep at the right time, whereas the homeostatic process determines the time and quality of sleep, and the body need to sleep when facing sleep deprivation (Donlea *et al*., 2014; Cavanaugh *et al*., 2016; Dubowy *et al*., 2016). Circadian rhythms are “built‐in” so that, without time signals from the environment, they keep time at the rate of about 24‐h periods. It is important that the rhythms be re‐set regularly to the natural light/dark cycle (Zheng & Sehgal, 2012). We found that *Wolbachia* did not disturb the circadian rhythm and sleep rebound after deprivation of *D. melanogaster* hosts (Bi *et al*., 2018). This suggests that *Wolbachia* infection does not break this internal rhythm under the regular 24 h clock and normal environment.

To explore the mechanisms of sleep behavior change, our further experiments showed that the interference of sleep caused by *Wolbachia* infection may be related to the dopamine pathway, as the two key genes in dopaminergic signaling pathway, *Pale* and *Ddc*, are both upregulated in *Wolbachia*‐infected flies compared to uninfected ones (Bi *et al*., 2018). Wu *et al*. (2018) showed that juvenile hormone (JH) signaling pathway was important for maintenance of sexually dimorphic sleep by regulating sex‐relevant genes, such as *fruitless* (*fru*) and *doublesex* (*dsx*) in males and *sex‐lethal* (*sxl*), *transformer* (*tra*) and *doublesex* (*dsx*) in females. Several studies have revealed that *Wolbachia* infection may make sterile *Sxl* mutant female hosts capable of producing mature eggs (Ote *et al*., 2016), and *Wolbachia* can also interfere with *dsx* gene expression in their insect host, such as expressing the female‐specific isoform of *dsx* in genetical males (Sugimoto *et al*., 2010). These suggest that *Wolbachia* may interact with sex‐relevant genes in their insect hosts. Dalton *et al*. (2013) identified that *dopamine receptor 2* was upregulated in response to overexpression of male‐specific *fru*, indicating that sex‐relevant genes may regulate the dopamine pathway. Furthermore, JH Inducible‐21 (JHI‐21) has been reported to be required for sleep/wake regulation, since downregulation of *JhI‐21* in dopaminergic neurons reduced the sensitivity to L‐DOPA, thus leading to an increase in daily sleep and sleep bout duration during the night. (Aboudhiaf *et al*., 2018). Correspondingly, our previous work found that *Wolbachia* could stimulate the JH signaling pathway in male *Drosophila* (Liu *et al*., 2014). Hence, it is reasonable that *Wolbachia* could affect sleep behavior of *Drosophila* through the JH/sex‐relevant gene/dopamine pathway.

Most animals would reduce activity or increase sleep when they get infections, which may therefore be considered as a general reflection of infection (Adelman & Martin, 2009; Kuo *et al*., 2010; Kuo & Williams, 2014). *Wolbachia* do not have the complete set of metabolic pathways present in free‐living bacteria (Wu *et al*., 2004). Studies have indicated that *Wolbachia* could affect host metabolic pathways to obtain the nutrients and energy for their own survival and proliferation (Brownlie *et al*., 2009; Evans *et al*., 2009; Yuan *et al*., 2015; Saucereau *et al*., 2017). Therefore it is reasonable to suggest that the increase of sleep in the hosts caused by *Wolbachia* could be an adaptive strategy to conserve limited resources and energy for reproductive output, which is in favor of both the maternally transmitted bacteria and the host. This could be the result of co‐evolution of *Wolbachia* and their hosts.

## Effect of *Wolbachia* on learning and memory capacity (LMC) in hosts

*Wolbachia* are known to accumulate in nervous tissues of their hosts, including the central brain. The central brain includes the antennal lobes, which are responsible for receiving input from the olfactory sensory neurons, and the mushroom bodies which provide sensory learning and memory capacities. Therefore, this distribution of *Wolbachia* raises the possibility that *Wolbachia* may influence host LMC. Indeed, some researchers showed that one kind of *Wolbachia*, *w*VulC could decrease the LMC of its host *Armadillidium vulgare*, a terrestrial isopod. *w*VulC‐infected *A. vulgare* are less likely to remember the correct direction after training when compared to the *Wolbachia*‐free group (Templé & Richard, 2015). This suggests that *Wolbachia* can affect cognitive processes in *A. vulgare*, resulting in a decrease of host adaptation. Kishani Farahani *et al*. (2017) reported that *Wolbachia* infection decreased the memory retention of host wasps in new environments relative to uninfected ones, thus increasing the spread of the endosymbiont through regulating wasps to forget the information about previous environments.

Recently, we found that *Wolbachia* infection significantly enhanced LMC in both *D. melanogaster* and *D. simulans* (Bi *et al*., 2019). This result is different from that reported by Templé and Richard (2015). We can interpret these opposite findings in two ways. (i) There could be differences in experimental design. They examined the effect of *Wolbachia* on short‐term memory (memory tested <1 h post‐training), while we tested at 24 h after training which is a long‐term memory. Moreover, they used only the chemical cues from the opposite sex as the “reward”, while we applied conditioning and testing protocols with sugar reward. (ii) It could be due to different *Wolbachia*/host combinations. Documents have revealed that different *Wolbachia* strains could induce distinct phenotypes in one host, and one *Wolbachia* strain may have various effects on different hosts (Dean, 2006; Chafee *et al*., 2011; Russell *et al*., 2018). In their case, *w*VulC *Wolbachia* is pathogenic and over‐replicates reducing the lifespan of its host. It also induces feminization of the host *A. vulgare* (Templé & Richard, 2015). While in our study, *w*Mel and *w*Ri *Wolbachia* are not pathogenic, they could induce sperm‐egg cytoplasmic incompatibility in *D. melanogaster* and *D. simulans*, respectively. Furthermore, the different taxonomic groups of the hosts (Crustacea and Insecta) may also contribute to the contrast results. *A. vulgare* have a gregarious lifestyle and a strong tendency for individuals to aggregate (Broly *et al*., 2013). This lifestyle may lead to a danger of being fully attacked by predators. The reduction of LMC in *A. vulgare* induced by *w*VulC *Wolbachia* may decrease the risk of being fully preyed upon, thus ensuring the survival and transmission of *Wolbachia*. However, *Drosophila* is solitary. The improved LMC caused by *Wolbachia* may be favorable for flies to find food and escape from dangerous environments. Evidence has suggested that LMC is likely to be important for fitness in a diverse set of taxa (Rice & McQuillan, 2018). For example, in grasshopper *Schistocerca americana*, insects that could employ associative learning had exhibited higher growth rates than insects that were prevented from learning (Dukas & Bernays, 2000). Similarly, in African striped mice *Rhabdomys pumilio*, higher spatial memory ability and faster reaction times are associated with increased survival rates (Maille & Schradin, 2016). From this aspect, the enhanced LMC due to *Wolbachia* infection could increase the fitness of the hosts and also benefit the survival and spread of the bacteria.

It remains largely unknown how *Wolbachia* influence the LMC of their hosts. We recently explored the molecular mechanisms and demonstrated that *Wolbachia* infection reduced the expression of a microRNA (miRNA) ─ *dme‐miR‐92b* in *D. melanogaster*, thus increased the expression of its target gene *crebA* in the head of flies by binding to the 3ʹ untranslated region (Bi *et al*., 2019). *CrebA* encodes cyclic‐adenosine monophosphate response element binding protein A and has been shown to have a role in dendrite development in *Drosophila* (Iyer *et al*., 2013). Evidence has shown that *CrebA* knockdown in *Drosophila* neurons affected dendritic terminals and total length, causing an overall reduction in branch complexity, indicating functions of *CrebA* in promoting higher order branching and dendritic growth (Iyer *et al*., 2013). Furthermore, other studies have revealed that changes in dendritic complexity impaired LMC (Freymuth & Fitzsimons, 2017; Lee *et al*., 2017). Both the microinjection of *dme‐miR‐92b* antagomirs and overexpression of *crebA* in *D. melanogaster* did result in significant increase of LMC (Bi *et al*., 2019), further supporting a miRNA‐target pathway that regulates the change of LMC in *Drosophila* induced by *Wolbachia* infection. Future investigations on the effects of *Wolbachia* infection and *crebA* mutation on fly dendrite development will further reinforce this argument.

The changes in host LMC could potentially contribute in evolutionary strategies. For the pathogenic strain of *Wolbachia*, the infection causes a decrease in host fitness. Thus the alteration in LMC could aim to avoid or slow down the parasites’ transmission in the population. However, for the *Wolbachia* strains that are not pathogenic, the adaptive alteration of the hosts induced by *Wolbachia* may be a kind of co‐evolution between the endosymbiont and their hosts. *Wolbachia* infection improves LMC in hosts which may help hosts to find food, mates and suitable habitats for offspring, and sometimes to avoid danger more efficiently. The benefits to the host also favor the proliferation and propagation of the maternal transmitted bacteria. These findings open a new approach for understanding host/endosymbionts interactions and in particular on *Wolbachia* impact on signal interpretation, learning and memory consequences in various hosts. As *Wolbachia*‐infected arthropods and nematodes could be intermediate hosts during infection of vertebrates, including humans, the behavioral and ecological consequences of arthropod infection should be taken into consideration while developing strategies for controlling pests and insect‐borne diseases.

## Effect of *Wolbachia* on the mating behavior of hosts

The role of *Wolbachia* in mediating mating behavior of their hosts remains controversial. Zhao *et al*. (2013) reported that in the two‐spotted spider mite *Tetranychus urticae*, *Wolbachia*‐infected and uninfected males did not show different mating behaviors when mated with uninfected females. Likewise, there is no evidence of male mate preferences in the butterfly *Acraea encedon* (Jiggins *et al*., 2002) and *D. innubila* (Sullivan & Jaenike, 2006), although *Wolbachia* can kill male offspring. However, numerous researchers have documented *Wolbachia* could influence the mating preferences, mating time, mating frequency of their hosts (de Crespigny *et al*., 2006; Panteleev *et al*., 2007; Goodacre & Martin, 2012). For example, de Crespigny *et al*. (2006) showed that infected males mate at a higher rate than uninfected males in both *D. melanogaster* and *D. simulans*. Additionally, the females of *D. paulistorum* semispecies show strong mating choice for intra‐semispecifc (homogamic) males harboring the same (compatible) symbiont variant. Such preference is lost when the titer of *Wolbachia* was significantly reduced in females, resulting in random mating with either *per se* incompatible, heterogamic males or compatible males (Miller *et al*., 2010). Correspondingly, the decrease of *Wolbachia* titer in male *D. paulistorum* led to a rejection of mating by homogamic wild‐type females (Schneider *et al*., 2019). This indicates that it is *Wolbachia* that are able to affect host mating behavior and thus can trigger pre‐ and post‐mating isolation. Furthermore, Fortin *et al*. (2018) showed that *A. vulgare* males spent more time near the *Wolbachia*‐free females than the *Wolbachia*‐infected females, when both females were virgin. This finding supported the result reported by Moreau *et al*. (2001) that *A. vulgare* males performed more insemination events with uninfected females. Consistently, Fortin *et al*. (2019) showed that *Wolbachia*‐infected *A. vulgare* females were less likely to be gravid in populations exhibiting an excess of females. Recently, we investigated the influence of *Wolbachia* infection on post‐mating responses (PMRs) of *D. melanogaster* and found that the male *Wolbachia* status had significant effect on the receptivity to remating of their mates. We observed that after mating with male *D. melanogaster* infected with *Wolbachia*, the females (regardless of the *Wolbachia* status) exhibited significantly decreased sexual receptivity to remating compared to those mated with uninfected males (Liu *et al*., 2014; He *et al*., 2018).

The different chemical profiles from female or male hosts according to the presence or absence of *Wolbachia* could explain the altered mating behavior caused by *Wolbachia*. In *Drosophila*, commensal bacteria altered the profile of cuticular hydrocarbons and influence mate choice (Sharon *et al*., 2010; Ringo *et al*., 2011). Recently, Schneider *et al*. (2019) have shown that *Wolbachia*‐knockdown *D. paulistorum* males express altered sexual pheromone profiles, with decrease between 9‐ and 23‐fold in several male‐specific compounds when compared to wild‐type levels. This is in accord with the finding in *A. vulgare*, where females presented variations in the relative proportion of chemical compounds according to the presence or the absence of *Wolbachia* (Richard, 2017; Fortin *et al.*, 2018). In many insects, sexual pheromones play an important role in recognition cues for mate choice between and within species. These suggest that *Wolbachia* might affect the mating behavior through alteration of some special sexual chemicals in their hosts. As to PMRs changed by *Wolbachia*, since insect seminal fluid proteins (Sfps) that are transferred to females with sperm during copulation have been demonstrated to play a role in modulating the physiological processes and behavior in the mated females (Avila *et al*., 2011; Denis *et al*., 2017), we assayed expressions of some genes coding for Sfps. Quantitative reverse‐transcription polymerase chain reaction (qRT‐PCR) analyses revealed that *Acp26Aa*, *CG1656*, and *CG42474* were significantly downregulated in *Wolbachia*‐infected males, compared to uninfected controls (He *et al*., 2018). By comparing the protein profiles of spermathecae and seminal receptacles from females mated with *Wolbachia*‐infected and uninfected male flies, we once identified a number of differentially expressed proteins, including some Sfps (Yuan *et al*., 2015). Most of them showed downregulation in the presence of *Wolbachia*. These suggest that *Wolbachia* may result in the changes in PMRs through changing the expressions of some Sfps in their insect hosts.

The altered mating behavior caused by *Wolbachia* might be a result of co‐evolution. That the infected males mate at a higher rate than uninfected males may increase the opportunity for *Wolbachia* propagation, or may be a behavioral adaptation employed by males to increase male reproductive success (de Crespigny *et al*., 2006). For those obligate *Wolbachia*–insect associations, such as the *D. paulistorum*–*Wolbachia* system, the changed mating bias in hosts is most likely for avoiding detrimental reproductive consequences like hybrid mortality, male sterility, producing descendants with decreased fitness (Ehrman, 1968; Kim & Ehrman, 1998; Moreau *et al*., 2002; Miller & Schneider, 2012). The altered female behavior and physiology after mating may be in favor of males while potentially hurting females (Chapman *et al*., 1995; Wigby & Chapman, 2005; Sirot *et al*., 2015; Smith *et al*., 2017). These opposed interests between the sexes can cause sexual conflict. However, females are not passive responders, instead they may utilize Sfps to produce physiological reactions as a trade‐off for mating costs. We have observed that female flies mated with *Wolbachia*‐infected males showed a significant decrease in sexual receptivity relative to those mated with uninfected males. This reaction may benefit female flies by reducing the costs of mating. This is supported by the enhanced median life span of the females mated with *Wolbachia*‐infected males compared to the mates of uninfected males (He *et al*., 2018). Hence, *Wolbachia* could modulate the sex conflicts of their insect hosts, and thus also favor *Wolbachia* because these bacteria can be transmitted only by female hosts. The observations that the female *D. paulistorum* semispecies prefer to mate with intra‐semispecifc (homogamic) males harboring the same (compatible) *Wolbachia* variant (Miller *et al*., 2010) also benefit the transmission of the bacteria.

## Effect of *Wolbachia* on feeding behavior of hosts

Many pathogens, such as dengue virus have to undergo a significant period of development in their insect vector before they can be transmitted to a new host, including humans. This indicates that only older insects are vectors of epidemiological importance. Hence, reducing mosquito lifespan has the potential to decrease disease transmission (Sinkins & O'Neill, 2000; McMeniman *et al*., 2009). One *Wolbachia* strain *w*Melpop, which can cause host cells being like a bag filled with popcorn due to its massive proliferation, has been found to shorten the lifespan of adult *Drosophila* by up to 50% (Min & Benzer, 1997), and thus has been first inducted to the mosquito vector *Aedes aegypti* (McMeniman *et al*., 2009). Mosquitoes carrying *w*MelPop‐CLA (cell‐line‐adapted) showed around a 50% reduction in lifespan of adults compared to uninfected mosquitoes (McMeniman *et al*., 2009). In addition, Turley *et al*. (2009) tested the feeding behavior of the mosquito and found that the presence of *w*MelPop‐CLA in *A. aegypti* did not affect the response time to humans, but reduced the number and size of blood meals taken. Especially in old mosquitoes, *w*MelPop‐CLA also led to reduced blood‐feeding success because when they tried to insert their stylets into a host skin their proboscises repeatedly bent. We recently compared the feeding behaviors between females after mating with *Wolbachia*‐infected and uninfected male *D. melanogaster*. We observed that females mated with infected males exhibited reduced feeding frequency compared to those mated with uninfected males, while the female *Wolbachia* status had no effects on their own feeding frequency (He *et al*., 2018), indicating that *Wolbachia*‐infected males could induce a notable reduction in food uptake of their mates.

The mechanisms by which *Wolbachia* affect the feeding behavior of insect hosts are not well known. To investigate whether the alterations in feeding behaviors caused by *w*MelPop‐CLA were due to the manipulation of neuroamines, Moreira *et al*. (2011) detected the expression of genes involved in the dopamine biosynthetic pathway and measured the amount of dopamine in mosquitoes. They did find *Wolbachia*‐infected mosquitoes have higher dopamine levels in their heads, but the effect was not present at all ages. Unexpectedly, there is no obvious correlation between higher dopamine levels and the abnormal behavioral phenotypes “shaky” and “bendy” shown by the older mosquitoes. Considering that in their original host *D. melanogaster*, *w*MelPop undergo massive proliferation in the adult, resulting in widespread degeneration of tissues, including brain and muscle, leading to early death (Min & Benzer, 1997), the altered feeding behavioral phenotypes “bendy” proboscis in mosquitoes is likely due to the degeneration of neurons caused by over‐proliferation of *w*MelPop. As to *Wolbachia*‐infected males inducing a decreased feeding frequency of their mates, this could be regulated by the Sfps derived from *Wolbachia*‐infected males as discussed above for PMRs.

Blood‐feeding on humans is just the time when pathogens like dengue virus are transmitted, hence damages in feeding behavior can decrease the transmission rate of viruses. *Wolbachia*‐infected mosquitoes are less able to obtain blood meals especially in old age. In addition, *Wolbachia* may interfere in transinfected mosquitoes with a wider range of pathogens including nematodes (Kambris *et al*., 2009), and dengue and Zika viruses (Moreira *et al*., 2009; Aliota *et al*., 2016a). Therefore, the World Health Organization has recommended the utilization of *Wolbachia* to counter the growing problem to human health caused by mosquito‐transmitted pathogens (WHO, 2016).

## Effect of *Wolbachia* on locomotive and aggressive behavior of hosts

It was reported that *Wolbachia* affected locomotion in *Drosophila* in response to olfactory cues, although the effects vary according to host background or environmental conditions (Peng *et al*., 2008; Peng & Wang, 2009; Caragata *et al*., 2011). Some studies have shown that *Wolbachia* infection may increase the locomotor activity of their insect hosts (Fleury *et al*., 2000; Evans *et al*., 2009; Peng & Wang, 2009). For example, the *w*Ri *Wolbachia* can increase the basal activity level of its native host *D. simulans*. Similarly, transinfection with the virulent *w*MelPop strain from *D. melanogaster* into *A. aegypti* results in an increase in locomotor activity in the mosquito (Evans *et al*., 2009). In contrast, *w*Mel and *w*MelPop were not able to cause any alterations in the basal activity levels of their native host *D. melanogaster*, although they cause slight decreases in responsiveness to food cues (Peng *et al*., 2008). In addition, our previous work also demonstrated that under laboratory conditions, *w*Ri‐infected *D. simulans* increased both locomotive activity and olfactory responsiveness (Peng & Wang, 2009) when compared to those uninfected controls. Moreover, we found that the olfactory responsiveness of the flies positively correlate to *Wolbachia* density in *D. simulans*, that is, the higher the *Wolbachia* density is in the flies, the faster the hosts’ olfactory responsiveness is, showing shorter time it took to be captured by the food trap. qRT‐PCR showed that the expression of an important odorant receptor gene *or83b* was significantly upregulated in flies with fast olfactory response relative to those with slow olfactory response (Peng & Wang, 2009).

Rohrscheib *et al*. (2015) once compared the male aggression in *D. melanogaster* infected with different strains of *Wolbachia*. They observed that *w*MelPop infection caused a significantly reduced initiation of aggressive encounters in male flies when compared against the behavior of the uninfected controls. But once starting, the duration of a fighting bout was comparable to that of uninfected control males. However, for the other two *Wolbachia* strains, *w*Mel and *w*MelCS, they did not exhibit any effects on *D. melanogaster* male aggression. Further analyses showed that the octopamine biosynthesis pathway was significantly downregulated in *w*MelPop‐infected male flies, which could explain the reduction in aggressive behavior (Rohrscheib *et al*., 2015). Since male aggression behavior is critically important in mate competition, the decreased male aggression caused by wMelPop infection may reduce the fitness in their hosts. Furthermore, wMelPop has a larger fecundity cost as well as other potential costs associated with lifespan reduction, feeding and probing behavior, quiescent eggs (in a dried state the wMelPop‐infected eggs tend to lose viability over time) (Nguyen *et al*., 2015). Thus the established *w*MelPop‐CLA infected *A. aegypti* mosquitoes are not applicable for biocontrol because they displayed reduced fitness in field releases, while *w*Mel‐infected mosquito lines were successfully established and released, and have been demonstrated to be effective at blocking local insect‐borne disease transmission (Walker *et al*., 2011; Hoffmann *et al*., 2014; Aliota *et al*., 2016a, 2016b; O'Neill *et al*., 2018; Achee *et al*., 2019).

## Perspectives

**Figure 1:**
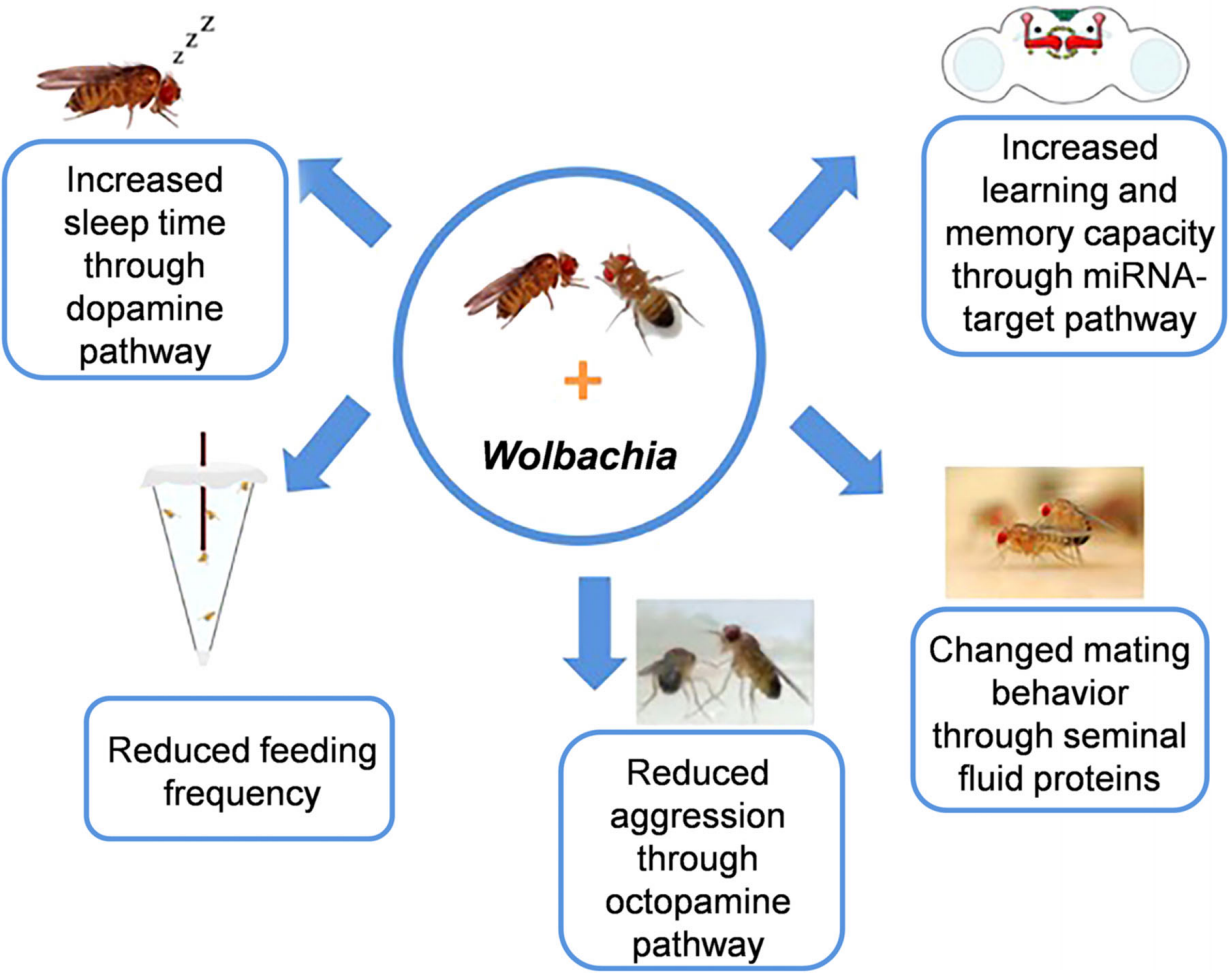
A simple model of the effects of *Wolbachia* infection on the behavior of insect hosts.

*Wolbachia* bacteria are best known for their ability to manipulate host reproduction, despite their widespread distributions in somatic tissues, including brain, and consequently they have the potential to affect host behaviors. Recently, extensive studies have contributed to our understanding the influences of the bacteria on insect behaviors, including sleep, learning and memory, mating, feeding, locomotion and aggression (Fig. 1). With the practice of *Wolbachia* in controlling insect‐borne diseases, more attention should be paid to the effects of *Wolbachia* on hosts’ behaviors so as to evaluate the influences on the environment of releasing these *Wolbachia*‐infected insects into the fields. However, because there are various inherent difficulties to dissect complex behavior, most of the behavioral studies were performed to date under the laboratory conditions. Questions about what influence *Wolbachia* infection has on the host behavior in field conditions and consequently on the ecosystem remain to be further studied. The molecular mechanisms by which *Wolbachia* affect host behaviors need to be more thoroughly and deeply elucidated. The effect of *Wolbachia* on host fitness traits may be multidimensional, and a number of host genes, miRNAs and proteins were modified by *Wolbachia* infection. As the development of technologies of bioinformatics and molecular biology continues, identifying effector molecules and their functional pathways associated with *Wolbachia* and their influence on host behaviors is also a main priority.

## Disclosure

The authors declare no conflicts of interest.
